# P-1218. Assessment of Cefepime-Taniborbactam (FEP-TAN) Transmembrane Clearance (CL_TM_) in an *Ex vivo* Continuous Renal Replacement Therapy (CRRT) Model

**DOI:** 10.1093/ofid/ofae631.1400

**Published:** 2025-01-29

**Authors:** Aliaa Fouad, Cole McGrath, Ecem Buyukyanbolu, Joseph L Kuti

**Affiliations:** Hartford Hospital, Farmington, Connecticut; Hartford Hospital, Farmington, Connecticut; Hartford Hospital, Farmington, Connecticut; Hartford Hospital, Farmington, Connecticut

## Abstract

**Background:**

Optimal antibiotic dosing in patients undergoing CRRT is influenced by many factors that affect drug adsorption and clearance. These factors include filter type, CRRT mode, effluent rate (ER), and drug characteristics. FEP-TAN is an investigational β-lactam/β-lactamase inhibitor combination having broad-spectrum activity against carbapenem-resistant Enterobacterales and *Pseudomonas aeruginosa*. Both FEP and TAN are primarily eliminated through glomerular filtration. Hence, we conducted an *ex vivo* CRRT model to characterize the adsorption and CL_TM_ when co-administered.

**Methods:**

CL_TM_ was characterized in hemofiltration (CVVH) and hemodialysis (CVVHD) modes using the Prismaflex ST150 and HF1400 hemofilter sets. One liter of heparinized bovine blood was allowed to circulate in the CRRT system for 10 minutes. FEP and TAN were then added simultaneously to attain plasma concentrations of 70 and 20 mg/L, respectively, the average C_max_ achieved in humans following FEP-TAN 2g/0.5g q8h as a 4h infusion. Pre-filter blood, post-filter blood, and effluent samples were collected over 60 minutes to determine FEP and TAN concentrations. Combinations of filters, modes, and points of fluid replacement were tested at ERs of 2, 3, and 4 L/h. The sieving coefficient (SC) for CVVH and the saturation coefficient (SA) for CVVHD were calculated to determine CL_TM_. Protein binding (PB) was assessed by ultrafiltration. Adsorption was determined using a closed circuit bypassing effluent elimination.

**Results:**

Adsorption and degradation in the *ex vivo* CRRT model were negligible for both drugs. Mean ± SD initial FEP and TAN concentrations were 66.9 ± 5.3 and 18.7 ± 1.7 mg/L, respectively. PB was 0% for FEP and 5.4 ± 3.7% for TAN in bovine plasma. The overall mean ± SD SC/SA across different modes, filters, and ERs were 1.1 ± 0.06 for FEP and 1.0 ± 0.05 for TAN. The CL_TM_ at ERs of 2, 3, and 4 L/h were 2.1 ± 0.2, 2.9 ± 0.3, and 3.8 ± 0.5 for FEP and 1.9 ± 0.2, 2.7 ± 0.2, and 3.4 ± 0.3 L/h for TAN, respectively.

**Conclusion:**

FEP and TAN were efficiently cleared by both CVVH and CVVHD CRRT modes through ST150 and HF1400 filters. When incorporated into an established population pharmacokinetic models, these data can be used to develop dosing recommendations for FEP-TAN in patients undergoing CRRT.

**Disclosures:**

**Joseph L. Kuti, PharmD**, Abbvie: Advisor/Consultant|bioMerieux: Grant/Research Support|Merck: Grant/Research Support|Pfizer: Grant/Research Support|Shionogi Inc: Advisor/Consultant|Shionogi Inc: Grant/Research Support|Shionogi Inc: Honoraria|Venatorx: Grant/Research Support

